# Complete genome of *Streptococcus agalactiae* strain XM_1

**DOI:** 10.1128/mra.00328-24

**Published:** 2024-09-05

**Authors:** Yingzhu Guo, Yao Zhu, Xinzhu Lin, Dai Wang

**Affiliations:** 1 Xiamen Key Laboratory of Perinatal-Neonatal Infection, Department of Neonatology, Women and Children’s Hospital, School of Medicine, Xiamen University, Xiamen, China; 2 State Key Laboratory of Vaccines for Infectious Diseases, Xiang An Biomedical Laboratory, School of Public Health, Xiamen University, XIamen, China; 3 State Key Laboratory of Molecular Vaccinology and Molecular Diagnostics, National Innovation Platform for Industry-Education Integration in Vaccine Research, Shenzhen Research Institute of Xiamen University, Shenzhen, China; University of Maryland School of Medicine, Baltimore, Maryland, USA

**Keywords:** *Streptococcus agalactiae*, complete genome

## Abstract

*Streptococcus agalactiae* can cause a variety of human diseases, posing a deadly threat especially in the case of infections in newborns. A strain of *Streptococcus agalactiae*, designated as XM_1, was isolated from a newborn with severe clinical symptoms. Here, the genome sequence of *Streptococcus agalactiae* strain XM_1 is presented.

## ANNOUNCEMENT

*Streptococcus agalactiae* is a pathogen causing a variety of human infections (1). In particular, its infection can be fatal for newborns, leading to pneumonia, meningitis, and sepsis (2). Strain XM_1 was isolated according to a standard protocol (3) from the cerebrospinal fluid of a newborn who exhibited critical clinical symptoms including meningitis and septicemia at Xiamen Women and Children’s Hospital in 2020.

Genomic DNA was extracted from bacterial pellet (Todd-Hewitt Broth, 37°C, 200 rpm) using Wizard Genomic DNA Purification Kit (Promega) and quantified for further analysis. Whole-genome sequencing was performed using PacBio Sequel IIe and Illumina sequencers (NovaSeq 6000). For Illumina library construction, genomic DNA was fragmented by Covaris and used agarose gel electrophoresis to choose fragments (~400 bp). Illumina sequencing libraries were prepared from the sheared fragments using the NEXTFLEX Rapid DNA-Seq Kit (Bioo Scientific). The prepared libraries then were used for paired-end Illumina sequencing (2 × 150 bp). For Illumina sequencing, the read length is 150 bp, and the number of raw pair reads is 2 × 3,677,689. By removing adapter sequences, trimming off 5´ ends containing non A, G, C, and T bases, trimming low-quality bases at the end of reads (sequencing quality value <Q20), discarding reads with a proportion of N bases reaching 10%, and discarding short fragments with a length <25 bp, a total of 3,602,883 paired clean reads were obtained.

For PacBio sequencing, genomic DNA was fragmented by G-tubes and used agarose gel electrophoresis to choose fragments at 8 ~ 10 kb. DNA fragments were then purified, end-repaired, and ligated with SMRTbell sequencing adapters (Pacific Biosciences). Next, the PacBio library was prepared by SMRTbell prep kit 3.0 (Pacbio) and sequenced on one SMRT cell using standard methods. Generally, a cutting parameter is determined based on the integrity of the extracted sample. During the DNA library construction, a round of bead purification for size selection will be conducted during the final purification step to remove fragments <5 kb. For PacBio sequencing, the number of raw reads is 30,995, and the number of read N50 is 109,00 bp.

The data generated from PacBio and Illumina platform were used for bioinformatics analysis. The raw Illumina sequencing reads were subjected to quality filtering using fastp v.0.20.0 (4). The HiFi reads were generated from the PacBio platform for analysis. Then, the clean short reads and HiFi reads were assembled to construct complete genomes using Unicycler v.0.4.8 (5) and Pilon v.1.22 (6) to polish the assembly using short-read alignments, reducing the rate of small errors. Default parameters were used except where otherwise noted.

The assembled genome was 2,108,309 bp in length, comprising one chromosome (1,971,498 bp, GC content: 35.41%) encoding 1,901 predicted coding sequences (CDS) and one plasmid (136,811 bp, GC content: 37.65%) encoding 170 predicted CDSs. Based on third-generation reads mapping, the genome coverage reached 151.42×. For circular genomes, when using the Unicycler software for assembly, Unicycler will automatically perform operations such as trimming and correction on the data, and the Flye v.2.9 (7) is used for self-assembly to remove overlapping regions. Default parameters were used except where otherwise noted.

## Data Availability

The raw reads were submitted to the NCBI SRA under accession number SRR28172286 and SRR28172285, BioProject accession number PRJNA1082423, and BioSample accession number SAMN40210270. The assembled data were submitted to the NCBI WGS under accession number CP147645-CP147646.
